# Supplementary material for: Mast Cells Differentiated in Synovial Fluid and Resident in Osteophytes Exalt the Inflammatory Pathology of Osteoarthritis
Source: Int J Mol Sci. 2022 Jan 4;23(1):541. doi: 10.3390/ijms23010541 (PMC8745477; doi:10.3390/ijms23010541)
Supplement: Supplementary file 1 [file ijms-23-00541-s001.zip › ijms-1486774-supplementary.pdf]

Title - Mast cells differentiated in synovial fluid and resident in osteophytes exalt the inflammatory pathology of osteoarthritis

Priya Kulkarni[1], Abhay Harsulkar[1,2], Anne-Grete Märtson[3], Siim Suutre[4], Aare Märtson[5,6] and Sulev Koks[7,8*]

[1]Department of Pathophysiology, Institute of Biomedicine and Translational medicine, University of Tartu, Ravila 19, Tartu- 50411, Estonia

[2]Department of Pharmaceutical Biotechnology, Poona College of Pharmacy, Bharati Vidyapeeth University, Erandwane, Pune – 411038, India

[3]Department of Pharmacology and Therapeutics, University of Liverpool, UK

[4]Department of Anatomy, Institute of Biomedicine and Translational medicine, University of Tartu, Ravila 19, Tartu- 50411, Estonia

[5]Department of Traumatology and Orthopaedics, Institute of Clinical Medicine, University of Tartu, L Puusepa 8, Tartu- 51014, Estonia

[6]Clinic of Traumatology and Orthopaedics, Tartu University Hospital, L Puusepa 8, Tartu- 51014, Estonia

[7]Perron Institute for Neurological and Translational Science, Nedlands, 6009, Western Australia

[8]Centre for Molecular Medicine and Innovative Therapeutics, Murdoch University, Murdoch, 6150, Western Australia

Supplementary table 1 (ST1): A list of significantly up-regulated and down-regulated genes in the osteophyte samples with their LogFC and *P*-values

**Up-regulated Genes**		
**Gene Name**	**LogFC**	***P*-value**
CPA3	4.027533	5.09E-18
SELE	2.536014	2.14E-16
MS4A2	4.220766	3.78E-15
PLA2G2A	4.644088	5.87E-12
CSN1S1	5.942081	1.44E-10
HAPLN1	4.354046	1.48E-10
GABRA4	3.303431	4.35E-10
PRG4	2.714763	5.14E-10
THBS4	2.993005	6.09E-10
IBSP	2.006641	7.31E-10
SLC36A2	2.247509	7.09E-09
HPGD	3.213818	9.49E-09
OGN	2.890213	4.86E-08
ASPN	2.432588	6.50E-08
CTSG	6.380478	7.17E-08
F5	2.485848	1.88E-07
FAM38B	2.125555	4.49E-07
IL1RL1	2.520738	8.72E-07
ZIC1	4.85166	1.12E-06
PRSS35	2.469275	1.73E-06
STMN2	2.835148	1.70E-06
MMP-13	3.19846	2.01E-06
CRTAC1	2.740265	2.06E-06
SHOX2	2.027773	7.05E-06

ACP5	2.83225	7.15E-06
COMP	3.324406	9.58E-06
COL1A2	2.01073	1.29E-05
LRFN5	3.088649	1.65E-05
TMEM196	2.776174	1.94E-05
HBA2	2.960499	2.08E-05
CTSK	2.240189	2.15E-05
CKB	2.25553	2.41E-05
GNG4	2.377001	3.22E-05
ADCYAP1	2.582899	3.33E-05
CNR1	2.254559	5.24E-05
ST18	2.889322	5.83E-05
MMP3	3.539804	6.33E-05
FZD10	2.231317	0.000133
GJB2	3.455437	0.000135
CYP27C1	4.37209	0.000173
C1orf186	3.272109	0.000199
HDC	2.110747	0.000243
CMA1	5.007723	0.000274
COL1A1	2.038679	0.000368
SYT6	2.129815	0.000422
AMPH	2.072226	0.000523
AMTN	3.814246	0.000729
TNFSF11	2.405489	0.001057
GALNT14	4.471982	0.001105
TRIM11	2.051431	0.001203
NELL1	4.426061	0.001209
TIMD4	2.79137	0.001212
HHIP	3.320885	0.001394
MRGPRX2	4.517302	0.001406
GFPT2	2.213461	0.001616
NPHS1	3.499433	0.001877
RASL12	2.011284	0.002044
HEMGN	2.173148	0.002365

CXCL9	2.226117	0.002528
IDO1	3.382825	0.002929
MYO3A	2.28746	0.00321
ADAM23	3.193211	0.003252
LRRC8E	2.234257	0.003983
SERPINA5	2.802678	0.004112
GYPA	2.738816	0.004161
SLC4A1	4.885617	0.004197
LIF	3.908902	0.004473
OPCML	2.428177	0.004786
PART1	2.41668	0.004784
CXCL10	2.225288	0.006315
FAM40B	2.022329	0.00656
C21orf37	4.084623	0.006829
WFDC1	2.136605	0.006992
KIF4A	2.248998	0.007723
FAM133A	2.053699	0.008372
CLDN10	2.516074	0.009417
SLC9A2	2.165385	0.010866
TUBA8	2.401238	0.010872
CXCL11	2.190709	0.013103
SULT1B1	3.152541	0.013765
MMP1	3.027436	0.014302
CILP	2.148754	0.014409
CRLF1	3.817562	0.015141
CA1	2.451945	0.017428
MARCO	3.779762	0.018039
LINGO1	2.318577	0.018117
C10orf105	2.176865	0.0222
**Down-regulated Genes**		
**Gene Name**	**LogFC**	**P-value**
APOB	-2.03918	9.05E-11

CADM2	-2.95074	1.41E-08
TMEFF2	-4.15435	0.000413
GNAZ	-3.41417	0.000625
GABRA2	-2.77263	0.000654
NRG3	-4.14112	0.001502
CHIT1	-3.25043	0.003048
C6orf10	-3.72459	0.003143
NETO1	-2.91119	0.00346
FAM86DP	-3.6999	0.00405
LOC100129345	-3.6983	0.004298
GLIS1	-3.69735	0.004474
IGSF1	-2.84473	0.004914
TRAF2	-3.68328	0.006547
SERPINA12	-2.98392	0.007455
TINAGL1	-3.55897	0.007825
LOC148824	-3.56945	0.009136
C1orf74	-3.55768	0.009161
FLJ30403	-3.40428	0.015644
LOC653113	-3.43474	0.018687
PIAS4	-3.40745	0.020407
PRINS	-3.40161	0.021045

ST2: A Grade-wise demonstration of key mast-related proteins found in the proteomics analysis of SF

Protein Name	Protein Abbreviation	LogFC G2G1	LogFC G3G1	LogFC G4G1	Ref
Immunoglobulin lambda variable 2-18	IGLV2-18	37.5896545	NA	37.42546	
Immunoglobulin lambda variable 3-1	IGLV3-1	36.59979569	NA	NA	
Immunoglobulin kappa variable 2-40	IGKV2-40	37.05100621	41.67871	37.29651	
Immunoglobulin lambda variable 2-11	IGLV2-11	2.431556072	2.782218	NA	
Immunoglobulin superfamily containing leucine-rich repeat protein	ISLR	NA	39.41972	NA	
Immunoglobulin kappa variable 1-13	IGKV1-13	NA	37.90953	NA	
Immunoglobulin kappa variable 6-21	IGKV6-21	NA	36.9165	NA	
Immunoglobulin lambda variable 4-69	IGLV4-69	NA	2.773235	NA	
Immunoglobulin superfamily containing leucine-rich repeat protein	ISLR	NA	NA	41.52395	
Immunoglobulin lambda variable 1-44	IGLV1-44	NA	NA	36.97934	
Immunoglobulin kappa variable 1-13	IGKV1-13	NA	NA	36.86832	
Immunoglobulin lambda variable 4-69	IGLV4-69	NA	NA	2.380682	
Protein S100 (Fragment)	S100A6	41.16882	40.40297	42.1267	
Protein S100-A8	S100A8	38.80698	43.95978	43.62165	
Protein S100-A9	S100A9	38.33136	44.35842	43.4431	
Protein S100-A4	S100A4	3.127559	-37.9506	2.521137	
Protein S100-A11	S100A11	0.747533	3.450775	4.473638	
Protein S100-A12	S100A12	NA	43.6355	44.17231	
Protein S100-P	S100P	NA	42.32842	41.86717	
Histone H3.1	H3C1	42.15141	43.86723	42.13659	

Histone H1.2	H1-2	40.8021	NA	40.73825	
Isoform 1 of Core histone macro-H2A.1	MACROH2A1	40.35853	41.30319	40.28084	
Histone H2A type 2-B	H2AC21	38.82677	40.98707	NA	
Histone H2A type 2-C	H2AC20	37.47739	38.57285	36.1769	
Histone H1.10	H1-10	36.92155	36.63726	36.05325	
Histone H1.5	H1-5	4.143112	1.894718	3.143245	
Histone H4	H4C1	4.135902	5.062983	3.918993	
Histone H3.2	H3C15	3.952981	4.763897	3.338169	
Histone H2A	H2AZ2	3.513314	38.20615	4.071692	
Histone H2B	H2BC15	2.290555	4.26651	2.707146	
Methionine aminopeptidase 2	METAP2	35.78272	33.33631	35.87134	
Xaa-Pro aminopeptidase 1	XPNPEP1	34.63523	37.59244	NA	
Aminopeptidase B	RNPEP	2.703409	5.132456	5.127756	
Alpha-actinin-1	ACTN1	40.01584	44.90512	44.6893	
Actin-related protein 2/3 complex subunit 3	ARPC3	39.73583	42.46596	42.05718	
Actin-related protein 3	ACTR3	2.494164	4.897881	4.0529	
Alpha-actinin-4	ACTN4	2.359276	4.658768	4.330454	
Actin-related protein 2	ACTR2	2.279216	4.53598	3.858559	
Actin, cytoplasmic 1	ACTB	2.240241	-37.5188	2.776209	[59]
Beta-actin-like protein 2	ACTBL2	NA	37.29973	35.91144	[59]
Platelet-activating factor acetylhydrolase IB subunit beta (Fragment)	PAFAH1B2	36.83481	35.17532	NA	
Ras-related protein Rab-7a	RAB7A	37.22036	39.44037	38.90048	[59]
Heat shock cognate 71 kDa protein	HSPA8	1.609628	1.574592	1.596362	[58]
Filamin-A	FLNA	3.974561	4.215035	3.994411	
Carboxypeptidase	CTSA	35.9543	NA	35.5287	
Carboxypeptidase Q	CPQ	1.294259	2.359289	-36.5255	
Cathepsin L1	CTSL	37.15384	36.42123	NA	
Cathepsin D	CTSD	2.680759	1.436841	3.760144	
Cathepsin G	CTSG	-37.5698	5.721166	5.100312	

Dual specificity mitogen-activated protein kinase kinase 1	MAP2K1	38.16372	38.24167	36.92404	
Mitogen-activated protein kinase 1	MAPK14	NA	39.43511	39.30365	
Mitogen-activated protein kinase 14	MAPK14	NA	39.48305	38.15754	
Mitogen-activated protein kinase	MAPK3	NA	36.16989	36.2431	
Phospholipase A2	PLA2G2A	2.003637	2.129905	0.998775	

ST3: Demographic details of OA patients from whom the osteophytes and control tissue samples were collected for RNA-seq analysis

**Patient No.**	**Demographic details**
**Patient 1**	Age: 76 years<br>Sex: male<br>BMI: 23.78<br>KL grade: 4
**Patient 2**	Age: 67 years<br>Sex: female<br>BMI: 35<br>KL grade: 4
**Patient 3**	Age: 69 years<br>Sex: male<br>BMI: 27.7<br>KL grade: 4
**Patient 4**	Age: 73 years<br>Sex: female<br>BMI: 32.4<br>KL grade: 4
**Patient 5**	Age: 52 years<br>Sex: female<br>BMI: 35<br>KL grade: 3
**Patient 6**	Age: 71 years<br>Sex: female<br>BMI: 29.1<br>KL grade: 4

ST4: Demographic details of OA patients from whom the SF samples were obtained and subjected to proteomics analysis and cell differentiation assay; SF samples obtained from Patient 1 to Patient 12 were used in *in vitro* cell differentiation assay, while the samples from Patient 1to Patient 16 were used for proteomics analysis

KL grade-I	KL grade-II	KL grade-III	KL grade-IV
Patient 1<br>Age – 53 years<br>Sex – male<br>BMI – 28.1	Patient 2<br>Age – 55 years<br>Sex – male<br>BMI – 30.1	Patient 3<br>Age – 50 years<br>Sex – female<br>BMI – 18.5	Patient 4<br>Age – 59 years<br>Sex – male<br>BMI – 27.1
Patient 5<br>Age – 40 years<br>Sex – female<br>BMI – 28.1	Patient 6<br>Age – 47 years<br>Sex – male<br>BMI – 25.3	Patient 7<br>Age – 55 years<br>Sex – female<br>BMI – 33.6	Patient 8<br>Age – 77 years<br>Sex – female<br>BMI – 25.5
Patient 9<br>Age – 70 years<br>Sex - female<br>BMI – 25.4	Patient 10<br>Age – 67 years<br>Sex - female<br>BMI – 27.5	Patient 11<br>Age – 68 years<br>Sex - female<br>BMI – 38.5	Patient 12<br>Age – 70 years<br>Sex - male<br>BMI – 21.1
Patient 13<br>Age – 65 years<br>Sex - male<br>BMI – 27.5	Patient 14<br>Age – 65 years<br>Sex - male<br>BMI – 26.2	Patient 15<br>Age – 48 years<br>Sex - male<br>BMI – 28.7	Patient 16<br>Age – 77 years<br>Sex - female<br>BMI – 22.6
**Average age**: 57 years<br>**Average BMI:** 27.28	**Average age**: 54.25 years<br>**Average BMI:** 27.28	**Average age**: 55.25 years<br>**Average BMI:** 29.83	**Average age**: 70.75 years<br>**Average BMI:** 24.08

SD1: Patient selection criteria for knee replacement surgery

Inclusion criteria: a confirmed OA diagnosis on the basis of clinical signs and symptoms as well as radiological evaluation with KL grade-III and IV and painful daily routine activities like walking, sitting, stair climbing; varus and valgus deformity; age between 40 to 80 years

Exclusion criteria: patients with rheumatoid arthritis, infected arthritis, KL grade- III and IV OA patients with tumour, KL grade-III and IV OA patients unfit for surgery due to chronic cardiovascular, renal and liver diseases.

SD2: OA patient selection criteria for arthrocentesis

OA patients with KL grades – I, II and III, who had knee effusion and required arthrocentesis to relieve the symptoms; OA patients with KL grade-IV who were selected for knee replacement surgery (as per the selection criteria mentioned in **SD1**)

All the patients, who were selected for SF collection, signed a written informed consent before the procedure.
